# Temperature extremes and infant mortality in Bangladesh: Hotter months, lower mortality

**DOI:** 10.1371/journal.pone.0189252

**Published:** 2018-01-05

**Authors:** Olufemi Babalola, Abdur Razzaque, David Bishai

**Affiliations:** 1 Department of International Health, Johns Hopkins Bloomberg School of Public Health, Baltimore, MD, United States of America; 2 International Centre for Diarrhoeal Disease Research Bangladesh (ICDDR,B), Dhaka, Bangladesh; 3 Department of Population, Family, and Reproductive Health, Johns Hopkins Bloomberg School of Public Health, Baltimore, MD, United States of America; The Ohio State University, UNITED STATES

## Abstract

**Background:**

Our study aims to obtain estimates of the size effects of temperature extremes on infant mortality in Bangladesh using monthly time series data.

**Methods:**

Data on temperature, child and infant mortality were obtained for Matlab district of rural Bangladesh for January 1982 to December 2008 encompassing 49,426 infant deaths. To investigate the relationship between mortality and temperature, we adopted a regression with Autoregressive Integrated Moving Average (ARIMA) errors model of seasonally adjusted temperature and mortality data. The relationship between monthly mean and maximum temperature on infant mortality was tested at 0 and 1 month lags respectively. Furthermore, our analysis was stratified to determine if the results differed by gender (boys versus girls) and by age (neonates (≤ 30 days) versus post neonates (>30days and <153days)). Dickey Fuller tests were performed to test for stationarity, and since the time series were non-stationary, we conducted the regression analysis based on the first differences of mortality and temperature.

**Results:**

Hotter months were associated with lower infant mortality in Bangladesh. Each degree Celsius increase in mean monthly temperature reduced monthly mortality by 3.672 (SE 1.544, p<0.05) points. A one degree increase in mean monthly temperature one month prior reduced mortality by 0.767 (SE 0.439, p<0.1) for boys and by -0.0764 (SE 0.366, NS) for girls. Beneficial effects of maximum monthly temperature were on the order of 0.623 to -0.712 and statistically significant for girls and boys respectively. Effect sizes of mean monthly temperature were larger for neonates at 1.126 (SE 0.499, p<0.05) than for post-neonates at 0.880 (SE 0.310, p<0.05) reductions in mortality per degree.

**Conclusion:**

There is no evidence that infant survival is adversely affected by monthly temperature extremes in Bangladesh. This may reflect a more heightened sensitivity of infants to hypothermia than hyperthermia in this environment.

## Introduction

Mounting scientific evidence supports the fact that human produced greenhouse gases are having an effect on the global climate and will likely lead to more extreme weather phenomena in the future. For example, the fourth assessment of the Intergovernmental Panel on Climate Change (IPCC) predicts that acute temperatures and precipitation are likely to increase in South Asia [1]. Studies of temperature and human mortality in the general population in cities in Europe, US, and China have shown a link [2–5]. In particular, studies have confirmed the negative effects of hot/cold temperatures on daily mortality [6–9]. Furthermore, this mortality risk varies by several factors including socio economic, cause of death, location and age [6, 10–13].

Studies on the effect of temperature extremes on mortality have been conducted mainly in developed countries where the frail population is mostly elderly [6, 10]. In contrast for low income populations, the group experiencing higher mortality includes infants and children as well as the elderly. It is unclear whether results obtained in the general population in high resource cities also apply to rural populations in low income countries.

Bangladesh is a very poor, low lying coastal region, with a history of flooding and extreme rainfall events, as well as droughts. The susceptibility of Bangladesh to frequent temperature extremes and the presence of a large population of rural children susceptible to heightened mortality make it a suitable setting to investigate the temperature-mortality relationship among low income children.

Our study will estimate the magnitude of temperature extremes on infant mortality in Bangladesh. We also stratify our analysis by neonatal/post-neonatal and by gender. Unlike previous literature exploring the temperature—mortality relationship in Bangladesh, this project employs the autoregressive integrated moving average (ARIMA) approach to adjust for autocorrelation [14]. ARIMA modelling has the advantage of isolating the direct and indirect effects of weather extremes on infant mortality by exploiting the associations in lagged relationships that exist within periodically collected data.

## Materials and methods

### Setting

This study was conducted in Matlab which is approximately 50km from Dhaka, the capital of Bangladesh. The area is a low–lying deltaic plain intersected by many rivers and canals and is typical of many rural and riverine areas of Bangladesh [15]. Farming is the dominant occupation, except in a few villages where fishing is the means of livelihood. Most of the farmers are in marginal situations with less than two acres of land, and 40% of them are landless. For many families, sharecropping and working on other people’s land on a daily-wage basis have become the main sources of livelihood.

### Data

Monthly infant and child mortality data (under 5 years) were available from the International Centre for Diarrhoeal Disease Research Bangladesh (ICDDR, B), which has been maintaining a Health and Demographic surveillance system in Matlab covering over 200,000 people since 1966. From this data, we also collected mortality information by gender (boys versus girls), and by age (neonates ≤30 days versus post neonates >30 days and < 153 days) for the period January 1982 to December 2008. Detailed temperature data (mean and maximum monthly temperatures) were also sourced from the Bangladesh Meteorological Department for the period January 1982 until December 2008. As a first step, all mortality and temperature data were tested using the Ljung–Box (Q) statistics to investigate autocorrelation, thereby confirming they were time series variables. Both temperature and mortality data were seasonally adjusted using monthly dummy variables. See S1 File.

### Analytical approach

The regression with Autoregressive Integrated Moving Averages (ARIMA) errors approach was used to examine the relationship between temperature and infant mortality. This method simply combines ordinary least squares and ARIMA models to correct for serial correlation often present in the error term of regressions involving time series variables [16]. The general structure of the model is as follows:

$$y_{t}=b_{1}x_{1,t}+\ldots \ldots .+b_{k}\;x_{k,t}+n_{t}$$ (1)

Where *n*_*t*_ is assumed to follow an ARIMA model. When *y*_*t*_ and *x*_*t*_ are differenced once, the combined regression with ARIMA (1,1,1) errors model is:

$${y^{\prime }}_{t}=b_{1}{x^{\prime }}_{1,t}+\ldots \ldots .b_{k}\;{x^{\prime }}_{k,t}+{n^{\prime }}_{t}$$

$$(1-\emptyset_{1}\;L){n^{\prime }}_{t}=(1+\theta_{1}\;L)e_{t}$$ (2)

Where *y*′_*t*_ = *y*_*t*_ − *y*_*t*−1_, *x*′_*t,i*_ = *x*_*t,i*_ − *x*_*t*−1_, *n*′_*t*_ = *n*_*t*_ − *n*_*t*−1_ and *e*_*t*_ is a white noise series [17].

The first step was to regress infant mortality on mean temperatures and check the residuals for stationarity and serial correlation. Stationarity in the residual series was investigated by both visual inspection and the application of the Augmented Dickey Fuller (ADF) test to a maximum lag of 16 as set by the Schwert criterion. The Box and Jenkins 3 step approach was used to fit an ARIMA model correcting for resultant serial correlation. We first induced stationarity in the residuals series by differencing both the dependent and explanatory variables. Secondly, after establishing stationarity of the differenced series and serial correlation in the regression residuals, sample autocorrelation and partial autocorrelation functions plots were used to identify the appropriate AR and MA orders of the ARIMA process. Finally, competing specifications were estimated and the Akaike Information criterion was used for model selection. See S5 and S6 Tables. Chosen models were also checked for adequacy using QQ plots and the Ljung Box test for serial correlation.

Mean temperature has been observed to be the best predictor of the temperature-mortality relationship when compared with other measures of temperature [18, 19]. However, we repeated the entire ARIMA fitting procedure using maximum monthly temperature as the predictor variable to investigate the mortality risk to infants due to extreme temperatures. Also, this temperature (mean and maximum)—mortality relationship was investigated at current temperature and one-month previous temperature i.e. 0 and 1 month temperature lag respectively. This approach is consistent with other studies that investigated the mortality effects of temperature extremes using a single lag [12, 20].

We explored the confounding effects of gender and age on the temperature- mortality relationship in this cohort of children. Our regression with ARIMA approach was repeated using specific mortality data for each age group or gender of children to see if the results differed by gender (boys versus girls] and age (neonates versus post-neonates). In total, we estimated 20 regressions with ARIMA models to explore this temperature-mortality relationship in Bangladesh.

## Results

### Descriptive analysis

Table 1 summarizes temperature and mortality statistics by age and gender over the study period. From 1982–2008, there were 49,426 recorded under-five child deaths with an average of 153 deaths per 1000 live births per month. Of these 49,426 deaths before age 5, there were 4,725 girls and 5,459 boys who died at ages less than 5 months old. The mean monthly neonate (<30 day) mortality was 23 deaths per 1000 births. Also, monthly mean temperature was 25.71°C on average while the mean maximum temperature is 33.15°C.

**Table 1:** Summary statistics of variables used in the analysis.

Variables	Mean	SD	Min.	Max
Mean temperature	25.71	2.76	21.80	34.70
Maximum	33.15	2.58	26	37.8
temperature				
Child mortality	152.55	42.25	83	382
age< 5 years				
Female-mortality	14.58	8.67	1	42
age <153 days				
Male mortality	16.85	9.33	0	60
age< 153 days				
Mortality	22.86	12.58	2	72
age≤30days				
Mortality	8.58	6.63	0	34
30days<age<153 days				

All data are monthly and temperature data is in °C. N = 323 monthly observations. Mortality is always measured as (monthly death count) 12 per 1000 live births in that calendar.

### Regression models with ARIMA errors

After differencing variables, stationarity in residuals was confirmed visually and with ADF tests up to the maximum lag of 16 for all relationships that were explored. The ARIMA fitting process also produced adequate models that removed most serial correlation left in the chosen models. Table 2 below shows the key statistics for the relationships investigated in this study. The significant coefficients of the temperature- mortality relationship were all negative. A 1°C increase in monthly mean temperature is associated with a reduction of 3.672 (SE 1.544, p<0.05) under 5 deaths per 1000. The beneficial effects of mean temperature were larger for males at 1.423 (SE 0.461, p<0.05) than females at 0.692 (SE 0.387, p<0.1) fewer deaths per degree Celsius. Beneficial effects of higher temperature were larger for neonates at 1.126 (SE 0.499, p<0.05) than for infants age 30 days to 153 days at 0.880 (SE 0.31, p<0.01).

**Table 2:** Relationships between mortality (under 5, female and male <153days, kids< = 30days and kids>30 days) and monthly temperature (mean and maximum) over lags 0 to 1 month.

Mortality					
	Child-mortality (Under 5)	Female-mortality (<153 days)	Male-mortality (<153 days)	Neonate mortality (<30 days)	Mortality between 30 and 153 days
**Regressors**					
**Mean temperature**					
Lag in months:					
0	-3.672**	-0.692*	-1.423***	-1.126**	-0.880***
	-(1.544)	-(0.387)	-(0.461)	-(0.499)	-(0.310)
1	**1.629**	**-0.076**	-0.767*	**-0.755**	**0.045**
	-(1.501)	-(0.366)	-(0.439)	-(0.483)	-(0.354)
ARIMA errors (lag = 0)	2,1,3	2,1,3	2,1,3	2,1,3	2,1,3
Ljung-Box Q statistic [6]	3.6	5.78	6.62	2.019	12.703
ARIMA errors (lag = 1)	1,1,1	2,1,3	2,1,3	2,1,3	2,1,3
Ljung-Box [6]	5.2	4.433	4.367	1.862	9.96
**Max temperature**					
Lag in months:					
0	**-1.36**	-0.712**	-0.623*	-1.137***	**-0.165**
	-(1.188)	-(0.329)	-(0.356)	-(0.409)	-(0.217)
1	**0.505**	**-0.194**	**-0.272**	**-0.352**	**-0.161**
	-(1.241)	-(0.276)	-(0.331)	-(0.343)	-(0.274)
ARIMA errors (lag = 0)	1,1,2	3,1,3	2,1,3	2,1,3	2,1,3
Ljung-Box Q statistic [6]	4.895	1.957	5.992	3.112	12.026
ARIMA errors (lag = 1)	1,1,2	2,1,3	2,1,3	2,1,3	2,1,3
Ljung-Box [6]	3.243	4.447	4.307	1.506	0.133

Standard errors in parentheses; Regression coefficients are marked as bold. Full table with ARIMA coefficients in S1 and S3 Tables.; All Q statistics confirmed the residuals of estimated models were white noise.

***p<0.01

** p<0.05

*p<0.1*

Models that looked at the effects of temperature with a lag of one month did not show consistent beneficial effects and only demonstrated a statistically significant effect for males. Models that examined the effects of maximum, instead of mean, monthly temperature demonstrated effects that were of similar size to the effects of mean monthly temperature except that the association between maximum temperature and male mortality under 153 days less than half the magnitude at 0.623 (SE 0.356, p<0.1). See S2 and S4 Tables.

## Discussion

In this study, we found a protective association between monthly temperature and mortality in Matlab, Bangladesh during 1982 to 2008. It was noted that hotter months were associated with reduced mortality; conversely the seasonally adjusted mortality risk was higher in colder months. This result is consistent with other studies that have reported increased mortality risk at lower temperatures.

Lindeboom et al. found a significant reduction in infant mortality at higher temperatures relative to lower temperatures in the Matlab area[14]. Hashizume et al. also noticed that there was a marked increase in all-cause mortality at low temperatures, while there was no heat effect at any lags in Bangladesh [15]. In addition there is little or no evidence suggesting increased child mortality due to heat waves, although there are some cold related deaths for this age group [21, 22]. Furthermore, the risk of heat–related mortality has been seen to be lower in warmer geographical locations, and some have conjectured mechanisms of acclimatization [12, 15, 23].

Prior studies show that the mortality risks from temperatures extreme might differ by demographic and socioeconomic status [20, 24, 25]. In this study, temperature effects on child mortality did not vary greatly by age or gender from the general trend; hotter temperature led to lower mortality in both neonates and post-neonates. That children respond differently to heat is not surprising because there is decreased thermoregulatory reserve with increased age[22, 26]. Although the coefficient of temperature induced mortality was larger in boys relative to girls, some of our estimates lacked precision so we cannot assert with confidence that there is a statistically significant difference between boys and girls.

The literature on gender difference in the mortality-temperature relationship is conflicting. Bai et al. and others have suggested that males are more vulnerable than females to temperature extremes [10, 20]. In other studies, women were seen to be more susceptible relative to men. Basu et al. suggests there is no modifying effect of gender in the temperature-mortality association in California [7].

Existing research has shown that temperature extremes may have both an instant and delayed (lag) effect on mortality [18, 22, 27]. However, there is no consensus in the literature on choosing the optimal lag length. We adopted the approach by Bell et al. and estimated the mortality effects of temperature at lag 0–1. Of all the ten estimated regressions investigating the lag 1 month temperature effect on child mortality, only three showed a positive but non-significant temperature-mortality association. Although not conclusive, this result suggests that it might be worthwhile to further investigate the delayed effect of temperature on child mortality in Bangladesh, particularly as it has been reported elsewhere that shorter lag lengths might amplify the heat effects and reduce the cold effects on mortality [15, 28].

This study has a few limitations that are worth mentioning. Firstly, our data for temperature and mortality are from a single district and should not be generalized for all of Bangladesh. However, Matlab is very typical of areas susceptible to the effects of climate change in Bangladesh. These places are characterized by several rivers and a low lying delta plain, hence making Matlab suitable for our temperature extremes mortality study. Secondly, several studies have suggested that socioeconomic factors like health status; level of poverty, education etc. influence this mortality risk from temperature extremes [29]. Because this study was done at an ecological level, we did not adjust for micro-level socio-economic variables that could have a potential confounding or modifying effect on the temperature-mortality relationship.

## Conclusion

In conclusion, our results show that the mortality risk to children from extreme temperatures is higher at colder months and lower at hotter months. This suggests that climate change related public health interventions should focus on the cold season in tropical countries like Bangladesh. Other phenomena associated with climate change such as increased floods, precipitation and poverty should be investigated in future research since they may be longer term aspects of climate change that may explain infant & child mortality in Bangladesh.

## Supporting information

S1 Table Models of mean temperature effects on infant mortality. Monthly infant mortality (Deaths before 12 months per 1000) regressed on MEAN monthly temp and MEAN temp in the prior month. All models use first differences of all variables to correct for non- stationarity. ARIMA terms included to minimize AIC.
(DOCX)

S2 Table Models of maximum temperature on infant mortality. Monthly infant mortality (Deaths before 12 months per 1000) regressed on MAXIMUM monthly temp and MAXIMUM temp in the prior month. All models use first differences of all variables to correct for non-stationarity.
(DOCX)

S3 Table Models of mean temperature on neonatal and post neonatal mortality. Monthly neonatal mortality (Deaths before 1 month per 1000) and monthly post neonatal mortality (Deaths between 30 and 153 days) regressed on MEAN monthly temp temp and MEAN temp in the prior month. All models use first differences of all variables to correct for non- stationarity. ARIMA terms included to minimize AIC. Both sexes analysed together.
(DOCX)

S4 Table Models of maximum temperature on neonatal and post neonatal mortality. Monthly neonatal mortality (Deaths before 1 month per 1000) and monthly post neonatal mortality (Deaths between 30 and 153 days) regressed on maximum monthly temp. All models use first differences of all variables to correct for non- stationarity. ARIMA terms included to minimize AIC. Both sexes analysed together.
(DOCX)

S5 Table ARIMA AIC rankings for model residuals at lag 0. Akaike Information criteria of ARMA models at time lag = 0.
(DOCX)

S6 Table ARIMA AIC rankings for model residuals at lag 1. Akaike Information criteria of ARMA models at time lag = 1.
(DOCX)

S1 File Data used in the analysis of mortality and temperature from Matlab, Bangladesh. Monthly reports of temperature, maximum temperature, minumum temperature, infant mortality, female infant mortality, male infant mortality, male mortality less than 30 days, male mortality greater than 30 days, female mortality less than 30 days, female mortality greater than 30 days, both sex mortality less than 30 days, and both sex mortality greater than 30 days.
(XLSX)
