# Correction: Temperature extremes and infant mortality in Bangladesh: Hotter months, lower mortality

**DOI:** 10.1371/journal.pone.0216570

**Published:** 2019-05-01

**Authors:** Olufemi Babalola, Abdur Razzaque, David Bishai

In Table 1, values for the mean “Monthly under 5 mortality” variable and mean “Monthly death count(age≤30days)” variable are incorrect. Please see the complete, correct Table 1 here.

**Table 1:** Summary statistics of variables used in the analysis.

Variables	Mean	SD	Min	Max
Mean temperature	25.71	3.64	16.6	30.3
Maximum temperature	33.15	2.58	26	37.8
Monthly under 5 mortality	105.16	54.85	21.43	331.53
Female monthly death count(age <153 days)	14.58	8.67	1	42
Male monthly death count(age <153 days)	16.85	9.33	0	60
Monthly death count(age≤30days)	22.86	12.58	2	72
Monthly death count (30days<age<153 days)	8.58	6.63	0	34

All data are monthly and temperature data is in°C. N = 323 monthly observations. Child monthly (under 5) mortality is always measured as monthly death count times 12 divided by 1000 live births in that calendar year.

In Table 2, the values calculated for “Child-mortality (Under 5)” are incorrect. Please see the complete, correct Table 2 here.

**Table 2:** Relationships between mortality (under 5, female and male <153days, kids< = 30days and kids>30 days) and monthly temperature (mean and maximum) over lags 0 to 1 month.

	Mortality				
	Child-mortality (Under 5)	Female-mortality (<153 days)	Male-mortality (<153 days)	Neonate mortality (≤30 days)	Mortality between 30 and 153 days
**Mean temperature**					
Lag in months:					
0	-4.03***	-0.692*	-1.423***	-1.126**	-0.880***
	(1.373)	(0.387)	(0.461)	(0.499)	(0.310)
1	**1.556**	**-0.076**	-0.767*	**-0.755**	**0.045**
	(1.475)	(0.366)	(0.439)	(0.483)	(0.354)
ARIMA errors (lag = 0)	3,1,3	2,1,3	2,1,3	2,1,3	2,1,3
Ljung-Box Q Statistic [6]	0.85	5.78	6.62	2.019	12.703
ARIMA errors (lag = 1)	3,1.3	2,1,3	2,1,3	2,1,3	2,1,3
Ljung-Box [6]	0.936	4.433	4.367	1.862	9.96
**Max temperature**					
Lag in months:					
0	-2.696**	-0.712**	-0.623*	-1.137***	**-0.165**
	(1.255)	(0.329)	(0.356)	(0.409)	(0.217)
1	**1.023**	**-0.194**	**-0.272**	**-0.352**	**-0.161**
	(1.38)	(0.276)b	(0.331)	(0.343)	(0.274)
ARIMA errors (lag = 0)	3,1,3	3,1,3	2,1,3	2,1,3	2,1,3
Ljung-Box Q Statistic [6]	1.64	1.957	5.992	3.112	12.026
ARIMA errors (lag = 1)	1,1,2	2,1,3	2,1,3	2,1,3	2,1,3
Ljung-Box [6]	5.53	4.447	4.307	1.506	0.133

Standard errors in parentheses; Regression coefficients are marked as bold. Full table with ARIMA coefficients in S1, S2, S3 and S4; All Q statistics confirmed the residuals of estimated models were white noise.

***p<0.01

** p<0.05

*p<0.1*

There are errors in the S1 Table and its caption. The correct caption is: “S1 Table. Models of mean temperature effects on child mortality and gender mortality. Monthly under 5 mortality ratio ((U5MR); Deaths before 60 months of age per 1000 live births) or gender mortality (count of monthly deaths before 153 days) regressed on MEAN monthly temp and MEAN temp in the prior month. All models use first differences of all variables to correct for non- stationarity. ARIMA terms included to minimize AIC.” Please view the complete, correct S1 Table below.

There are errors in the S2 Table and its caption. The correct caption is: “S2 Table. Models of maximum temperature effects on child mortality and gender mortality. Monthly under 5 mortality ratio (Deaths before 60 months of age per 1000 live births) or gender mortality (count of monthly deaths before 153 days) regressed on MAXIMUM monthly temp and MAXIMUM temp in the prior month. All models use first differences of all variables to correct for non-stationarity.” Please view the complete, correct S2 Table below.

There are errors in the S3 Table caption. The correct caption is: “S3 Table. Models of mean temperature effects on neonatal and post neonatal. Monthly neonatal mortality (Death count before 1 month) and monthly post neonatal mortality (Death count between 30 and 153 days) regressed on MEAN monthly temp and MEAN temp in the prior month. All models use first differences of all variables to correct for non- stationarity. ARIMA terms included to minimize AIC. Both sexes analysed together.”

There are errors in the S4 Table caption. The correct caption is: “S4 Table. Models of maximum temperature effects on neonatal and post neonatal. Monthly neonatal mortality (Deaths before 1 month) and monthly post neonatal mortality (Death count between 30 and 153 days) regressed on MEAN monthly temp and MEAN temp in the prior month. All models use first differences of all variables to correct for non- stationarity. ARIMA terms included to minimize AIC. Both sexes analysed together.”

There are errors in the S5 Table and its caption. The correct caption is: “S5 Table. ARIMA AIC rankings for model residuals at lag 0. Table S5 below shows the AIC of several ARMA models fitted to the residuals (e1) of the various regressions at time lag = 0” Please view the complete, correct S5 Table below.

There are errors in the S6 Table and its caption. The correct caption is: “S6 Table. ARIMA AIC rankings for model residuals at lag 1. Table S6 below shows AIC of ARMA models fitted to the residuals (e2) of the various regressions at time lag = 1” Please view the complete, correct S6 Table below.

There are errors in S1 File. Please view the complete, correct S1 File below.

## Supporting information

S1 Table Models of mean temperature effects on child mortality and gender mortality. Monthly under 5 mortality ratio ((U5MR); Deaths before 60 months of age per 1000 live births) or gender mortality (count of monthly deaths before 153 days) regressed on MEAN monthly temp and MEAN temp in the prior month. All models use first differences of all variables to correct for non- stationarity. ARIMA terms included to minimize AIC.
(DOCX)

S2 Table Models of maximum temperature effects on child mortality and gender mortality. Monthly under 5 mortality ratio (Deaths before 60 months of age per 1000 live births) or gender mortality (count of monthly deaths before 153 days) regressed on MAXIMUM monthly temp and MAXIMUM temp in the prior month. All models use first differences of all variables to correct for non-stationarity.
(DOCX)

S3 Table Models of mean temperature effects on neonatal and post neonatal. Monthly neonatal mortality (Death count before 1 month) and monthly post neonatal mortality (Death count between 30 and 153 days) regressed on MEAN monthly temp and MEAN temp in the prior month. All models use first differences of all variables to correct for non- stationarity. ARIMA terms included to minimize AIC. Both sexes analysed together.
(DOCX)

S4 Table Models of maximum temperature effects on neonatal and post neonatal. Monthly neonatal mortality (Deaths before 1 month) and monthly post neonatal mortality (Death count between 30 and 153 days) regressed on MEAN monthly temp and MEAN temp in the prior month. All models use first differences of all variables to correct for non- stationarity. ARIMA terms included to minimize AIC. Both sexes analysed together.
(DOCX)

S5 Table ARIMA AIC rankings for model residuals at lag 0. Table S5 below shows the AIC of several ARMA models fitted to the residuals (e1) of the various regressions at time lag = 0
(DOCX)

S6 Table ARIMA AIC rankings for model residuals at lag 1. Table S6 below shows AIC of ARMA models fitted to the residuals (e2) of the various regressions at time lag = 1
(DOCX)

S1 File Data used in the analysis of mortality and temperature from Matlab, Bangladesh. Monthly reports of temperature, maximum temperature, minumum temperature, infant mortality, female infant mortality, male infant mortality, male mortality less than 30 days, male mortality greater than 30 days, female mortality less than 30 days, female mortality greater than 30 days, both sex mortality less than 30 days, and both sex mortality greater than 30 days.
(XLS)

## References

[1] Babalola O, Razzaque A, Bishai D (2018) Temperature extremes and infant mortality in Bangladesh: Hotter months, lower mortality. PLoS ONE 13(1): e0189252 10.1371/journal.pone.0189252 29304145 PMC5755750

