# Supplementary material for: Temperature extremes and infant mortality in Bangladesh: Hotter months, lower mortality
Source: PLoS One. 2018 Jan 5;13(1):e0189252. doi: 10.1371/journal.pone.0189252 (PMC5755750; doi:10.1371/journal.pone.0189252)
Supplement: S1 Table — Monthly infant mortality (Deaths before 12 months per 1000) regressed on MEAN monthly temp and MEAN temp in the prior month. All models use first differences of all variables to correct for non- stationarity. ARIMA terms included to minimize AIC. (DOCX) [file pone.0189252.s001.docx]

# **S1 Table. Models of mean temperature effects on children and infants****.** Monthly infant mortality (Deaths before 12 months per 1000) regressed on MEAN monthly temp and MEAN temp in the prior month. All models use first differences of all variables to correct for non- stationarity. ARIMA terms included to minimize AIC

| Model | A1 |  |  | B1 |  |  | C1 |  |
| --- | --- | --- | --- | --- | --- | --- | --- | --- |
|  | All Under 5 | All Under 5 | Female <153 |  | Female<153 | Male< 153 |  | Male < 153 |
|  |  |  | Days |  | Days | days |  |  |
| VARIABLES | Mortality | Mortality | Mortality |  | Mortality | Mortality |  | Mortality |
| Mean Monthly | -3.672** |  | -0.692* |  |  | -1.423*** |  |  |
| Temp | (1.544) |  | (0.387) |  |  | (0.461) |  |  |
| Mean Temp 1 |  | 1.629 |  |  | -0.0764 |  |  | -0.767* |
| month prior |  | (1.501) |  |  | (0.366) |  |  | (0.439) |
| L.ar | 1.566*** | 0.282** | 1.687*** |  | 1.700*** | 0.613*** |  | 0.601*** |
|  | (0.0740) | (0.121) | (0.0605) |  | (0.0614) | (0.121) |  | (0.117) |
| L2.ar | -0.578*** |  | -0.936*** |  | -0.955*** | -0.779*** |  | -0.699*** |
|  | (0.0742) |  | (0.0605) |  | (0.0656) | (0.171) |  | (0.161) |
| L.ma | -2.135*** | -0.825*** | -2.598*** |  | -2.562*** | -1.642*** |  | -1.620*** |
|  | (0.0131) | (0.101) | (0.106) |  | (0.110) | (0.0995) |  | (0.0930) |
| L2.ma | 1.374*** |  | 2.436*** |  | 2.393*** | 1.523*** |  | 1.445*** |
|  | (0.00331) |  | (0.198) |  | (0.211) | (0.214) |  | (0.194) |
| L3.ma | -0.239*** |  | -0.845*** |  | -0.822*** | -0.913*** |  | -0.854*** |
|  | (0.0159) |  | (0.0974) |  | (0.103) | (0.123) |  | (0.121) |
| Constant | -0.283*** | -0.280 | -0.0678*** |  | -0.0703*** | -0.0736*** |  | -0.0745*** |
|  | (0.0674) | (0.252) | (0.00775) |  | (0.00902) | (0.00736) |  | (0.00730) |
| Sigma | 18.00*** | 18.35*** | 4.313*** |  | 4.440*** | 4.642*** |  | 4.714*** |
|  | (0.902) | (0.975) | (0.222) |  | (0.203) | (0.279) |  | (0.301) |
| Observations | 323 | 322 | 323 |  | 322 | 323 |  | 322 |

Standard errors in parentheses; *** p<0.01, ** p<0.05, * p<0.1
