# Supplementary material for: Temperature extremes and infant mortality in Bangladesh: Hotter months, lower mortality
Source: PLoS One. 2018 Jan 5;13(1):e0189252. doi: 10.1371/journal.pone.0189252 (PMC5755750; doi:10.1371/journal.pone.0189252)
Supplement: S2 Table — Monthly infant mortality (Deaths before 12 months per 1000) regressed on MAXIMUM monthly temp and MAXIMUM temp in the prior month. All models use first differences of all variables to correct for non-stationarity. (DOCX) [file pone.0189252.s002.docx]

**S2 Table. Models of maximum temperature.** Monthly infant mortality (Deaths before 12 months per 1000) regressed on MAXIMUM monthly temp and MAXIMUM temp in the prior month. All models use first differences of all variables to correct for non-stationarity

| Model |  | A2 | B2 | C2 |  |
| --- | --- | --- | --- | --- | --- |
|  | All Under 5 | All Under 5 | Female Female<15 | Male< 153 | Male < 153 |
|  |  |  | <153 Days 3 Days | days |  |
| VARIABLES | Mortality | Mortality | Mortality Mortality | Mortality | Mortality |
| Max Monthly | -1.360 |  | -0.712** | -0.623* |  |
| Temp | (1.188) |  | (0.329) | (0.356) |  |
| Max Temp 1 |  | 0.505 | -0.194 |  | -0.272 |
| month prior |  | (1.241) | (0.276) |  | (0.331) |
| L.ar | 0.920*** | 0.734** | -1.449*** 1.702*** | 0.618*** | 0.598*** |
|  | (0.0292) | (0.374) | (0.111) (0.0617) | (0.0760) | (0.100) |
| L2.ar |  |  | -0.264 -0.956*** | -0.756*** | -0.667*** |
|  |  |  | (0.163) (0.0659) | (0.184) | (0.242) |
| L3.ar |  |  | 0.307*** |  |  |
|  |  |  | (0.0804) |  |  |
| L.ma | -1.526*** | -1.313*** | 0.734*** -2.633*** | -1.640*** | -1.558*** |
|  | (0.0742) | (0.404) | (0.0929) (0.118) | (0.0671) | (0.0744) |
| L2.ma | 0.526*** | 0.356 | -0.909*** 2.507*** | 1.513*** | 1.374*** |
|  | (0.0752) | (0.319) | (0.0779) (0.229) | (0.190) | (0.231) |
| L3.ma |  |  | -0.783*** -0.883*** | -0.911*** | -0.785*** |
|  |  |  | (0.122) (0.114) | (0.140) | (0.193) |
| Constant | -0.287*** | -0.302* | -0.0666*** -0.0702*** | -0.0755*** | -0.0756*** |
|  | (0.0524) | (0.165) | (0.00574) (0.00909) | (0.00875) | (0.00803) |
| Sigma | 18.21*** | 18.29*** | 4.451*** 4.287*** | 4.681*** | 4.847*** |
|  | (0.901) | (0.935) | (0.208) (0.227) | (0.303) | (0.288) |
| Observations | 323 | 322 | 323 322 | 323 | 322 |

Standard errors in parentheses; *** p<0.01, ** p<0.05, * p<0.1
