# Supplementary material for: Temperature extremes and infant mortality in Bangladesh: Hotter months, lower mortality
Source: PLoS One. 2018 Jan 5;13(1):e0189252. doi: 10.1371/journal.pone.0189252 (PMC5755750; doi:10.1371/journal.pone.0189252)
Supplement: S4 Table — Monthly neonatal mortality (Deaths before 1 month per 1000) and monthly post neonatal mortality (Deaths between 30 and 153 days) regressed on maximum monthly temp. All models use first differences of all variables to correct for non- stationarity. ARIMA terms included to minimize AIC. Both sexes analysed together. (DOCX) [file pone.0189252.s004.docx]

**S4 Table. Models of maximum temperature on neonatal and post neonatal mortality.**  Monthly neonatal mortality (Deaths before 1 month per 1000) and monthly post neonatal mortality (Deaths between 30 and 153 days) regressed on maximum monthly temp

All models use first differences of all variables to correct for non- stationarity. ARIMA terms included to minimize AIC. Both sexes analysed together.

| Model VARIABLES | D2  Neonatal <30 days | Neonatal | PostNeonatal | E2  PostNeonatal |
| --- | --- | --- | --- | --- |
|  |  | <30 days | (30 to 153 | (30 to 153 days) |
|  |  |  | days) |  |
| Max Monthly | -1.137*** |  | -0.165 |  |
| Temp | (0.409) |  | (0.217) |  |
| Max Temp 1 |  | -0.352 |  | -0.161 |
| month prior |  | (0.343) |  | (0.274) |
| L.ar | 1.722*** | 1.722*** | 1.697*** | 1.701*** |
|  | (0.0181) | (0.0193) | (0.0295) | (0.0289) |
| L2.ar | -0.989*** | -0.989*** | -0.959*** | -0.963*** |
|  | (0.0141) | (0.0152) | (0.0364) | (0.0355) |
| L.ma | -2.684*** | -2.686*** | -2.572*** | -2.576*** |
|  | (0.0473) | (0.0527) | (0.0696) | (0.0733) |
| L2.ma | 2.622*** | 2.624*** | 2.441*** | 2.447*** |
|  | (0.0755) | (0.0859) | (0.141) | (0.145) |
| L3.ma | -0.951*** | -0.951*** | -0.864*** | -0.866*** |
|  | (0.0328) | (0.0373) | (0.0713) | (0.0718) |
| Constant | -0.0953*** | -0.0969*** | -0.0509*** | -0.0514*** |
|  | (0.0156) | (0.0159) | (0.00322) | (0.00402) |
| Sigma | 5.310*** | 5.389*** | 3.482*** | 3.458*** |
|  | (0.277) | (0.292) | (0.206) | (0.203) |
| Observations | 323 | 322 | 323 | 322 |
