# Supplementary material for: Temperature extremes and infant mortality in Bangladesh: Hotter months, lower mortality
Source: PLoS One. 2018 Jan 5;13(1):e0189252. doi: 10.1371/journal.pone.0189252 (PMC5755750; doi:10.1371/journal.pone.0189252)
Supplement: S6 Table — Akaike Information criteria of ARMA models at time lag = 1. (DOCX) [file pone.0189252.s006.docx]

## S6 Table. ARIMA AIC rankings for model residuals at lag 1. Akaike Information criteria of ARMA models at time lag=1.

| ARM A | A1 | A2 | B1 | B2 | C1 | C2 | D1 | D2 | E1 | E2 |
| --- | --- | --- | --- | --- | --- | --- | --- | --- | --- | --- |
| 101 | **2796.369** | 2797.002 | 1903.88  9 | 1903.00  7 | 1956.16 | 1953.94  1 | 2099.08  8 | 2098.43  8 | 1739.52  5 | 1741.667 |
| 102 | 2796.565 | **2796.522** | 1905.223 | 1904.057 | 1958.272 | 1955.68 | 2096.431 | 2095.575 | 1743.325 | 1741.252 |
| 103 | 2797.73  4 | **2798.04**  **6** | 1906.87  8 | 1905.61  7 | 1950.60  1 | 1948.58  1 | 2093.2 | 2092.03  9 | 1744.69  2 | 1744.712 |
| 201 | 2797.01  8 | **2797.10**  **5** | 1905.03  7 | 1903.77 | 1949.84  7 | 1947.79  9 | 2092.67  9 | 2091.61  8 | 1743.29  4 | 1743.177 |
| 202 | 2798.31  6 | **2798.94**  **4** | 1906.98 | 1905.71  4 | 1951.811 | 1949.76  6 | 2094.25  9 | 2093.14  8 | 1744.17  9 | 1743.849 |
| 203 | 2798.52  8 | **2798.72**  **6** | 1893.93  8 | 1891.79  2 | 1949.63  7 | 1947.35  1 | 2053.60  2 | 2052.21  6 | 1732.36  7 | 1731.294 |
| 301 | 2799.00  8 | **2799.07**  **3** | 1906.89  4 | 1905.68 | 1951.78  7 | 1949.74  4 | 2093.96  9 | 2092.77 | 1745.13  4 | 1744.972 |
| 302 | 2796.41  7 | **2798.65**  **2** | 1901.85  3 | 1900.41  5 | 1952.00  7 | 1951.71  2 | - | 2093.51  6 | 1746.02  7 | 1745.758 |
| 303 | 2798.14  7 | **2797.49**  **2** | 1900.52  5 | 1898.54  5 | 1955.78  4 | 1950.10  9 | - | 2054.07  3 | 1744.36  3 | 1741.062 |
