# Supplementary material for: Correction: Temperature extremes and infant mortality in Bangladesh: Hotter months, lower mortality
Source: PLoS One. 2019 May 1;14(5):e0216570. doi: 10.1371/journal.pone.0216570 (PMC6493745; doi:10.1371/journal.pone.0216570)
Supplement: S1 Table — Monthly under 5 mortality ratio ((U5MR); Deaths before 60 months of age per 1000 live births) or gender mortality (count of monthly deaths before 153 days) regressed on MEAN monthly temp and MEAN temp in the prior month. All models use first differences of all variables to correct for non- stationarity. ARIMA terms included to minimize AIC. (DOCX) [file pone.0216570.s001.docx]

# **S1 Table. Models of mean temperature effects on child mortality and gender mortality.** Monthly under 5 mortality ratio ((U5MR); Deaths before 60 months of age per 1000 live births) or gender mortality (count of monthly deaths before 153 days) regressed on MEAN monthly temp and MEAN temp in the prior month. All models use first differences of all variables to correct for non- stationarity. ARIMA terms included to minimize AIC

| Model | A1 |  |  | B1 |  |  | C1 |  |
| --- | --- | --- | --- | --- | --- | --- | --- | --- |
| VARIABLES | U5MR_β_ | U5MR_π_ | Female Death Count <153 days Da | | | Male Death Count<153 days | | |
| Mean Monthly | -4.030*** |  | -0.692* |  |  | -1.423*** |  |  |
| Temp | (1.373) |  | (0.387) |  |  | (0.461) |  |  |
| Mean Temp 1 |  | 1.556 |  |  | -0.0764 |  |  | -0.767* |
| month prior |  | (1.475) |  |  | (0.366) |  |  | (0.439) |
| L.ar | -1.439*** | -1.430*** | 1.687*** |  | 1.700*** | 0.613*** |  | 0.601*** |
|  | (0.174) | (0.162) | (0.0605) |  | (0.0614) | (0.121) |  | (0.117) |
| L2.ar | -0.357 | -0.340 | -0.936*** |  | -0.955*** | -0.779*** |  | -0.699*** |
|  | (0.314) | (0.297) | (0.0605) |  | (0.0656) | (0.171) |  | (0.161) |
| L3.ar | 0.320* | 0.326** |  |  |  |  |  |  |
|  | (0.168) | (0.158) |  |  |  |  |  |  |
| L.ma | 0.971*** | -0.958*** | -2.598*** |  | -2.562*** | -1.642*** |  | -1.620*** |
|  | (0.163) | (0.15) | (0.106) |  | (0.110) | (0.0995) |  | (0.0930) |
| L2.ma | -0.453 | -0.462* | 2.436*** |  | 2.393*** | 1.523*** |  | 1.445*** |
|  | (0.283) | (0.259) | (0.198) |  | (0.211) | (0.214) |  | (0.194) |
| L3.ma | -0.796*** | -0.787*** | -0.845*** |  | -0.822*** | -0.913*** |  | -0.854*** |
|  | (0.153) | (0.137) | (0.0974) |  | (0.103) | (0.123) |  | (0.121) |
| Constant | -0.443 | -0.452 | -0.0678*** |  | -0.0703*** | -0.0736*** |  | -0.0745*** |
|  | (0.327) | (0.329) | (0.00775) |  | (0.00902) | (0.00736) |  | (0.00730) |
| Sigma | 19.85*** | 20.10*** | 4.313*** |  | 4.440*** | 4.642*** |  | 4.714*** |
|  | (1.088) | (1.105) | (0.222) |  | (0.203) | (0.279) |  | (0.301) |
| Observations | 323 | 322 | 323 |  | 322 | 323 |  | 322 |

Standard errors in parentheses; *** p<0.01, ** p<0.05, * p<0.1

Description of table S1 (above) has been updated to reflect data modification

β= new results in this column due to change in under 5 mortality data

π= new results in this column due to change in under 5 mortality data
