# Supplementary material for: Correction: Temperature extremes and infant mortality in Bangladesh: Hotter months, lower mortality
Source: PLoS One. 2019 May 1;14(5):e0216570. doi: 10.1371/journal.pone.0216570 (PMC6493745; doi:10.1371/journal.pone.0216570)
Supplement: S2 Table — Monthly under 5 mortality ratio (Deaths before 60 months of age per 1000 live births) or gender mortality (count of monthly deaths before 153 days) regressed on MAXIMUM monthly temp and MAXIMUM temp in the prior month. All models use first differences of all variables to correct for non-stationarity. (DOCX) [file pone.0216570.s002.docx]

**S2 Table. Models of maximum temperature effects on child mortality and gender mortality.** Monthly under 5 mortality ratio (Deaths before 60 months of age per 1000 live births) or gender mortality (count of monthly deaths before 153 days) regressed on MAXIMUM monthly temp and MAXIMUM temp in the prior month. All models use first differences of all variables to correct for non-stationarity.

| Model |  | A2 | B2 | C2 |  |
| --- | --- | --- | --- | --- | --- |
|  | U5MR_β_ | U5MR_π_ | Female Death Count <153 days | Male Death Count <153 days | |
|  |  |  |  |  |  |
| VARIABLES |  |  |  |  |  |
| Max Monthly | -2.696 |  | -0.712** | -0.623* |  |
| Temp | (1.255) |  | (0.329) | (0.356) |  |
| Max Temp 1 |  | 1.023 | -0.194 |  | -0.272 |
| month prior |  | (1.38) | (0.276) |  | (0.331) |
| L.ar | 1.475*** | 0.877*** | -1.449*** 1.702*** | 0.618*** | 0.598*** |
|  | (0.177) | (0.0537) | (0.111) (0.0617) | (0.0760) | (0.100) |
| L2.ar | -0.323 |  | -0.264 -0.956*** | -0.756*** | -0.667*** |
|  | (0.326) |  | (0.163) (0.0659) | (0.184) | (0.242) |
| L3.ar | 0.364** |  | 0.307*** |  |  |
|  | (0.176) |  | (0.0804) |  |  |
| L.ma | 1.023*** | -1.449*** | 0.734*** -2.633*** | -1.640*** | -1.558*** |
|  | (0.158) | (0.103) | (0.0929) (0.118) | (0.0671) | (0.0744) |
| L2.ma | -0.492* | 0.449*** | -0.909*** 2.507*** | 1.513*** | 1.374*** |
|  | (0.289) | (0.103) | (0.0779) (0.229) | (0.190) | (0.231) |
| L3b  .ma | -0.813*** |  | -0.783*** -0.883*** | -0.911*** | -0.785*** |
|  | (0.157) |  | (0.122) (0.114) | (0.140) | (0.193) |
| Constant | -0.44 | -0.491*** | -0.0666*** -0.0702*** | -0.0755*** | -0.0756*** |
|  | (0.33) | (0.0408) | (0.00574) (0.00909) | (0.00875) | (0.00803) |
| Sigma | 19.77*** | 20.25*** | 4.451*** 4.287*** | 4.681*** | 4.847*** |
|  | (1.09) | (1.049) | (0.208) (0.227) | (0.303) | (0.288) |
| Observations | 323 | 322 | 323 322 | 323 | 322 |

Standard errors in parentheses; *** p<0.01, ** p<0.05, * p<0.1

Description of table S2 (above) has been updated to reflect data modification

β= new results in this column due to change in under 5 mortality data

π= new results in this column due to change in under 5 mortality data
