# Supplementary material for: Correction: Temperature extremes and infant mortality in Bangladesh: Hotter months, lower mortality
Source: PLoS One. 2019 May 1;14(5):e0216570. doi: 10.1371/journal.pone.0216570 (PMC6493745; doi:10.1371/journal.pone.0216570)
Supplement: S3 Table — Monthly neonatal mortality (Death count before 1 month) and monthly post neonatal mortality (Death count between 30 and 153 days) regressed on MEAN monthly temp and MEAN temp in the prior month. All models use first differences of all variables to correct for non- stationarity. ARIMA terms included to minimize AIC. Both sexes analysed together. (DOCX) [file pone.0216570.s003.docx]

**S3 Table. Models of mean temperature effects on neonatal and post neonatal.** Monthly neonatal mortality (Death count before 1 month) and monthly post neonatal mortality (Death count between 30 and 153 days) regressed on MEAN monthly temp and MEAN temp in the prior month. All models use first differences of all variables to correct for non- stationarity. ARIMA terms included to minimize AIC. Both sexes analysed together.

| Model VARIABLES | D1  Neonatal <30 days | Neonatal | PostNeonatal | E1  PostNeonatal |
| --- | --- | --- | --- | --- |
|  |  | <30 days | (30 to 153 | (30 to 153 days) |
|  |  |  | days) |  |
| Mean Monthly | -1.126** |  | -0.880*** |  |
| Temp | (0.499) |  | (0.310) |  |
| Mean Temp 1 |  | -0.755 |  | 0.0450 |
| month prior |  | (0.483) |  | (0.354) |
| L.ar | 1.723*** | 1.723*** | 1.692*** | 1.702*** |
|  | (0.0190) | (0.0188) | (0.0278) | (0.0280) |
| L2.ar | -0.989*** | -0.989*** | -0.953*** | -0.964*** |
|  | (0.0150) | (0.0147) | (0.0330) | (0.0342) |
| L.ma | -2.684*** | -2.588*** | -2.576*** | -2.581*** |
|  | (0.0514) | (0.0446) | (0.0567) | (0.0682) |
| L2.ma | 2.618*** | 2.463*** | 2.447*** | 2.457*** |
|  | (0.0835) | (0.0726) | (0.115) | (0.135) |
| L3.ma | -0.947*** | -0.862*** | -0.871*** | -0.871*** |
|  | (0.0363) | (0.0332) | (0.0590) | (0.0672) |
| Constant | -0.0951*** | -0.0960*** | -0.0501*** | -0.0518*** |
|  | (0.0149) | (0.0151) | (0.00190) | (0.00370) |
| Sigma | 5.360*** | 5.655*** | 3.424*** | 3.460*** |
|  | (0.280) | (0.274) | (0.194) | (0.205) |
| Observations | 323 | 322 | 323 | 322 |

Description of S3 Table (above) has been modified to reflect proper description of data
