# Supplementary material for: Correction: Temperature extremes and infant mortality in Bangladesh: Hotter months, lower mortality
Source: PLoS One. 2019 May 1;14(5):e0216570. doi: 10.1371/journal.pone.0216570 (PMC6493745; doi:10.1371/journal.pone.0216570)
Supplement: S5 Table — Table S5 below shows the AIC of several ARMA models fitted to the residuals (e1) of the various regressions at time lag = 0 (DOCX) [file pone.0216570.s005.docx]

### **S5 Table. ARIMA AIC rankings for model residuals at lag 0.**

Table S5 below shows the AIC of several ARMA models fitted to the residuals (e1) of the various regressions at time lag= 0

| ARMA | A1 | A2 | B1 | B2 | C1 | C2 | D1 | D2 | E1 | E2 |
| --- | --- | --- | --- | --- | --- | --- | --- | --- | --- | --- |
| 101 | 2871.963 | 2872.695 | 1905.334 | 1906.223 | 1947.221 | 1957.391 | 2099.068 | 2097.984 | 1739.36 | 1747.132 |
| 102 | 2867.461 | 2866.107 | 1906.519 | 1907.27 | 1948.335 | 1958.859 | 2097.292 | 2095.726 | 1739.2 | 1750.947 |
| 103 | 2873.207 | 2873.579 | 1908.195 | 1908.339 | 1943.271 | 1951.2 | 2094.466 | 2091.15 | 1741.734 | 1749.228 |
| 201 | 2871.74 | 2871.80 | 1906.333 | 1906.868 | 1942.138 | 1950.407 | 2093.724 | 2091.18 | 1741.166 | 1748.9 |
| 202 | 2873.096 | 2873.485 | 1908.271 | 1908.614 | 1944.116 | 1952.395 | 2095.171 | 2092.104 | 1741.585 | 1751.204 |
| 203 | 2875.065 | 2875.478 | **1896.748** | 1910.339 | **1941.946** | **1949.896** | **2050.383** | **2046.502** | **1730.268** | **1740.943** |
| 301 | 2873.251 | 2873.547 | 1908.214 | 1908.349 | 1944.105 | 1952.387 | 2094.638 | 2091.14 | 1742.816 | 1752.39 |
| 302 | 2875.095 | 2875.485 | 1903.753 | 1902.785 | 1946.091 | 1952.157 | 2083.241 | 2078.101 | 1743.274 | 1752.845 |
| 303 | **2865.518** | **2865.838** | 1897.061 | **1901.353** | 1948.112 | - | 2052.111 | 2080.704 | 1741.806 | 1743.603 |

- - Bolded numbers represent minimum AIC for each relationship
  - Entries in A1 and A2 changed due to U5MR update
