# Supplementary material for: Correction: Temperature extremes and infant mortality in Bangladesh: Hotter months, lower mortality
Source: PLoS One. 2019 May 1;14(5):e0216570. doi: 10.1371/journal.pone.0216570 (PMC6493745; doi:10.1371/journal.pone.0216570)
Supplement: S6 Table — Table S6 below shows AIC of ARMA models fitted to the residuals (e2) of the various regressions at time lag = 1 (DOCX) [file pone.0216570.s006.docx]

### **S6 Table. ARIMA AIC rankings for model residuals at lag 1.**

Table S6 below shows AIC of ARMA models fitted to the residuals (e2) of the various regressions at time lag= 1

| ARM A | A1 | A2 | B1 | B2 | C1 | C2 | D1 | D2 | E1 | E2 |
| --- | --- | --- | --- | --- | --- | --- | --- | --- | --- | --- |
| 101 | 2869.173 | 2868.626 | 1903.889 | 1903.007 | 1956.16 | 1953.941 | 2099.088 | 2098.438 | 1739.525 | 1741.667 |
| 102 | 2865.38 | **2862.446** | 1905.223 | 1904.057 | 1958.272 | 1955.68 | 2096.431 | 2095.575 | 1743.325 | 1741.252 |
| 103 | 2870.547 | 2870.118 | 1906.878 | 1905.617 | 1950.601 | 1948.581 | 2093.2 | 2092.039 | 1744.692 | 1744.712 |
| 201 | 2869.213 | 2868.421 | 1905.037 | 1903.77 | 1949.847 | 1947.799 | 2092.679 | 2091.618 | 1743.294 | 1743.177 |
| 202 | 2870.457 | 2869.904 | 1906.98 | 1905.714 | 1951.811 | 1949.766 | 2094.259 | 2093.148 | 1744.179 | 1743.849 |
| 203 | 2872.36 | 2871.879 | **1893.938** | **1891.792** | **1949.637** | **1947.351** | **2053.602** | **2052.216** | **1732.367** | **1731.294** |
| 301 | 2871.042 | 2870.344 | 1906.894 | 1905.68 | 1951.787 | 1949.744 | 2093.969 | 2092.77 | 1745.134 | 1744.972 |
| 302 | 2872.431 | 2871.90 | 1901.853 | 1900.415 | 1952.007 | 1951.712 | - | 2093.516 | 1746.027 | 1745.758 |
| 303 | **2860.834** | 2865.02 | 1900.525 | 1898.545 | 1955.784 | 1950.109 | - | 2054.073 | 1744.363 | 1741.062 |

- - Bolded numbers represent minimum AIC for each relationship
  - Entries in A1 and A2 changed due to U5MR update
